# Follow-Up Liver Stiffness Measurements after Liver Resection Influence Oncologic Outcomes of Hepatitis-B-Associated Hepatocellular Carcinoma with Liver Cirrhosis

**DOI:** 10.3390/cancers11030425

**Published:** 2019-03-25

**Authors:** Jung Il Lee, Hyun Woong Lee, Seung Up Kim, Sang Hoon Ahn, Kwan Sik Lee

**Affiliations:** 1 Department of Internal Medicine, Yonsei University College of Medicine, Seoul 03722, Korea; LHWDOC@yuhs.ac (H.W.L.); KSUKOREA@yuhs.ac (S.U.K.); AHNSH@yuhs.ac (S.H.A.); LEEKS519@yuhs.ac (K.S.L.); 2 Gangnam Severance Hospital, Yonsei University College of Medicine, Seoul 06273, Korea; 3 Severance Hospital, Yonsei University College of Medicine, Seoul 03722, Korea

**Keywords:** hepatocellular carcinoma, liver cirrhosis, liver stiffness, dynamic changes, antiviral therapy, late recurrence

## Abstract

The severity of liver fibrosis can be noninvasively evaluated by measuring liver stiffness (LS) using transient elastography. This study aimed to evaluate the prognostic value of achieving low liver stiffness measurement (LSM) in patients with cirrhosis confirmed from the resected liver due to hepatocellular carcinoma (HCC). A total of 184 patients that received curative surgery for HCC related to the hepatitis B virus at Barcelona Clinic Liver Cancer stage 0–A, and had a METAVIR fibrosis score of 4 were investigated. LSM significantly decreased after antiviral therapy during follow-up (*p* = 0.001), and achieving LSM ≤8 kilopascal (kPa) suggested a reduced risk of late recurrence (>12 months) (hazard ratio (HR), 0.519; 95% confidence interval (CI), 0.307–0.877; *p* = 0.014). Older age at surgery (≥45 years) and multiple HCC nodules predicted an increased risk of late recurrence (HR, 3.270; 95% CI, 1.296–8.251; *p* = 0.012; and HR, 3.146; 95% CI, 1.396–7.089; *p* = 0.006). Decreased LSM also suggested decreased mortality (HR, 0.251; 95% CI, 0.086–0.756; *p* = 0.045) along with baseline low aspartate aminotransferase-to-platelet ratio index (APRI) score (<1.5) (HR, 0.251; 95% CI, 0.086–0.759; *p* = 0.041). Having early HCC recurrence (HR, 9.416; 95% CI, 3.566–24.861; *p* < 0.001) and microvascular tumor invasion (HR, 3.191; 95% CI, 1.188–8.568; *p* = 0.021) predicted increased mortality. Among HCC patients with liver cirrhosis under antiviral therapy, achieving low LSM (≤8 kPa) predicted reduced late HCC recurrence.

## 1. Introduction

Liver cirrhosis has been identified as the single most important factor for hepatocellular carcinoma (HCC) development [1]. It is also one of the most important prognostic factors after curative HCC treatment [2,3]. Meanwhile, studies demonstrate that prolonged antiviral therapy is associated with improvement in liver histology and reversal of cirrhosis in chronic infection with hepatitis B virus (HBV) and this might significantly change the prognosis of patients with HBV-related cirrhosis [4,5,6,7].

Although liver biopsy is considered the gold standard to diagnose the severity of fibrosis [8], it is an invasive procedure with a possible sampling error, which makes it almost impossible and unethical to perform sequentially. Instead, transient liver stiffness measurement (LSM), obtained by transient elastograpy (TE), is a noninvasive means of assessing liver fibrosis documented to be well correlated with biopsy-detected severity of fibrosis [9,10,11]. Studies reported that TE might be useful in not only detecting advanced liver fibrosis but also in stratifying the risk of HCC development in HBV patients [12,13,14]. It has been suggested that HBV patients with higher liver stiffness (LS) values (>8 kilopascal (kPa)) were at a significantly greater risk of HCC development [12]. However, dynamic changes in LSM can be achieved with antiviral therapy [15,16,17], and it has been recently reported that having LSM < 11.6 kPa after antiviral therapy was associated with a reduced risk of de novo HCC occurrence [18]. Although a decrease in LS values might be associated with decreased incidence of HCC, it has not been well documented whether antiviral therapy and reduction of LSM would have a beneficial effect on oncologic outcomes of HCC patients experiencing cancer development in a cirrhotic background. A study reported that background liver cirrhosis demonstrated a higher incidence of de novo recurrence of HCC [19], and it would be interesting to evaluate whether a reduction in LSM achieved with antiviral therapy would result in favorable prognosis in these patients.

It is not unusual to have LSM decrease as low as the point where advanced liver fibrosis is unlikely to be suspected after prolonged antiviral therapy in patients with clinically diagnosed liver cirrhosis [20,21]. However, a reduction in LS values may not necessarily indicate pathologic improvement [22], and the patients may still be at greater risk of HCC carcinogenesis due to the background liver cirrhosis.

Thus, this study evaluated impact of LS reduction to the point where advanced liver fibrosis was unlikely to be suspected using LSM criteria (LS ≤8 kpa), in HBV-related HCC patients whose pathological assessment at the time of HCC occurrence showed advanced liver fibrosis.

## 2. Patients and Methods

### 2.1. Patient Enrollment

This retrospective cohort study was conducted at a tertiary referral hospital in Seoul, Republic of Korea. A total of 1113 patients who underwent liver resection due to HBV-related HCC between January, 2007 and December, 2016 were screened for possible enrollment in this study. Among them, patients that met the following inclusion criteria were selected: curatively resected HBV-related HCC at Barcelona Clinic Liver Cancer (BCLC) stage 0–A; receiving antiviral therapy against HBV started at or after HCC resection for more than 12 months; available LSM, assessed at least 12 months after starting the antiviral therapy; METAVIR fibrosis score of 4, assessed from the resected liver. Exclusion criteria were as follows: receiving antiviral therapy started before the development of HCC; survival time of less than 4 weeks after the liver resection; co-infection with HIV or hepatitis C; previous history of liver transplantation. Finally, 184 patients were recruited for the final analysis. This study was performed in accordance with ethical guidelines of the 1975 Declaration of Helsinki and was approved by the Institutional Review Board of Gangnam Severance Hospital (Permit No: 3-2018-0198).

### 2.2. Baseline Workup and Follow-Up

The index date for study entry was defined as the time of liver resection. The studies performed within 3 months before the surgery were included in the baseline workup. Variables pertaining to HCC including tumor size, number, microvascular invasion, Edmonson grade, and METAVIR fibrosis score were obtained by pathologic evaluation of the resected liver. The patients were regularly followed-up. Liver function tests and alpha-fetoprotein (AFP) measurements were done every 3 months. Dynamic CT or dynamic MRI were regularly performed at intervals no longer than 6 months. As the inclusion criteria indicated, all the patients had LS measured more than once at least 12 months after staring the antiviral therapy. For those with multiple measurements, the data from the last study were considered as the follow-up results. Other biochemical measurements within 1 month before or after the last LS assessment were taken as the follow-up findings. All the patients received the antiviral therapy against HBV starting at or after the surgery for more than 12 months. HCC recurrence was classified as early (≤12 months) or late (>12 months) [23,24]. All the patients were followed until the time of death or for at least 12 months.

### 2.3. Assessment of Fibrotic Burden

All the patients were confirmed to have liver cirrhosis (METAVIR fibrosis score of 4) by pathologic assessment at the time of surgery. In order to determine changes in fibrotic burden by noninvasive means, serum biomarkers of liver fibrosis and LSM were used.

Serum biochemical markers of fibrosis, namely aspartate aminotransferase (AST)-to-platelet ratio index (APRI) [25], and Fibrosis (FIB)-4 [26], were calculated according to published formulae as follows.

APRI = ((AST/upper limit of normal)/platelet count (10^9^/L)) × 100

A cutoff value of APRI ≥1.5 was applied to detect a high probability of advanced fibrosis, as previously published [25].

FIB-4 = (Age (years) × AST (U/L))/(platelet count (10^9^/L) × (alanine aminotransferase (ALT) (U/L))^1/2^)

A cutoff value of FIB-4 ≥2.67 was used to detect intermediate and high probability of advanced fibrosis [26].

LSM was measured using transient elastography (TE) (FibroScan, EchoSens, Paris, France). Only LSM with at least 10 valid measurements, a success rate of at least 60%, and an interquartile range-to-median ratio <30% were considered reliable, following suggestions from previous studies [12,27]. In this study, LSM ≤8 kPa was considered as low fibrotic burden according to a previous study [12].

### 2.4. Statistical Analyses

Data were summarized as mean ± standard deviation (SD), median with range, or *n* (%), as appropriate. Categorical variables were compared using two-sided χ^2^-test (or Fisher’s exact test, or McNemar test, where appropriate) and continuous variables were compared using independent or paired sample *t*-tests (or Mann-Whitney test, where appropriate). Cumulative HCC recurrence rates and mortality rates were analyzed using Kaplan-Meier’s method and compared with the log-rank test. To identify independent risk factors of mortality and HCC recurrence, univariate and subsequent multivariate regression analyses were used. Hazard ratios (HRs) and corresponding 95% confidence intervals (CIs) were used where indicated.

All statistical analyses were performed using IBM SPSS (version 23). A *p*-value < 0.05 was considered to indicate statistical significance.

## 3. Results

### 3.1. Patient Selection and Demographic Findings

A total of 184 patients that received curative liver resection for HBV-related HCC at BCLC stage 0–A were investigated. The baseline demographic findings are described in Table 1 according to the HCC recurrence status classified into early (≤12 months) or late (>12 months) [23,24]. All the patients were followed until the time of death or for at least 12 months.

The patients that received low-barrier drugs (lamivudine, telbivudine, adefovir) eventually had the medication changed to high-barrier drugs (entecavir, tenofovir). None of the patients experienced biochemical breakthrough due to emergence of antiviral resistant mutation.

Baseline and follow-up values of the model for end-stage liver disease (MELD) score and serum biomarkers of liver fibrosis are compared in Table 2. Baseline values were calculated using biochemical variables measured at the time of the surgery and follow-up values were calculated using the results obtained at the time of the index follow-up LSM assessment.

**Figure 1:**
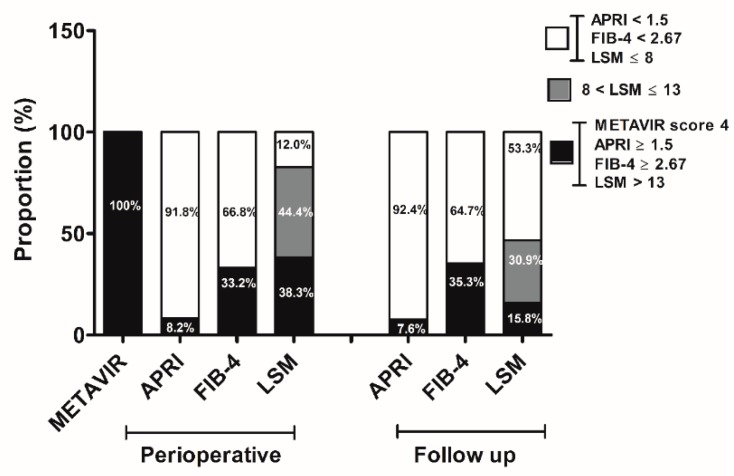
Changes in fibrotic burden. The proportion of patients with advanced fibrosis was assessed noninvasively by obtaining aspartate aminotransferase (AST)-platelet ratio index (APRI) and FIB-4 as well as liver stiffness (LS) values at the time of liver resection and during follow-up. METAVIR fibrosis score was evaluated from the resected liver at the time of HCC surgery.

Although all the patients had pathologically detected cirrhosis, advanced fibrosis was evident by APRI in 8.2% (15/184) and FIB-4 in 33.2% (61/184) of the patients at the time of liver resection. In addition, LSM >13 kPa, which would suggest liver cirrhosis, was shown in 38.3% (51/133), whereas LSM >8 kPa, which is reported to predict an increased risk of HCC development [12], was demonstrated in 82.7% (110/133) of the patients at baseline (Figure 1). Follow-up assessments after receiving antiviral therapy for more than 12 months demonstrated no significant changes in the proportion of advanced liver fibrosis by APRI (7.6%, 14/184) or FIB-4 (35.3%, 65/184) (*p* = 1.000, *p* = 0.742, respectively), whereas the proportion of high LSM (>8 kPa) significantly decreased (46.7%, 86/184) compared with that from the initial evaluation (*p* < 0.001) (Figure 1).

### 3.2. Follow-Up LSM as a Predictor of HCC Recurrence

The cumulative HCC recurrence-free survival rates (RFS) were 90.2%, 81.8%, 76.4%, and 66.8% at 1, 2, 3, and 5 years, respectively.

Late recurrence (>12 months) was detected in 58 patients (58/184, 31.5%). Older age at liver resection (≥45 year) (HR, 3.270; 95% CI: 1.296–8.251; *p* = 0.012) and multiple HCC nodules (HR, 3.146; 95% CI: 1.396–7.089; *p* = 0.006) suggested an increased risk of late recurrence (Table 3).

**Figure 2:**
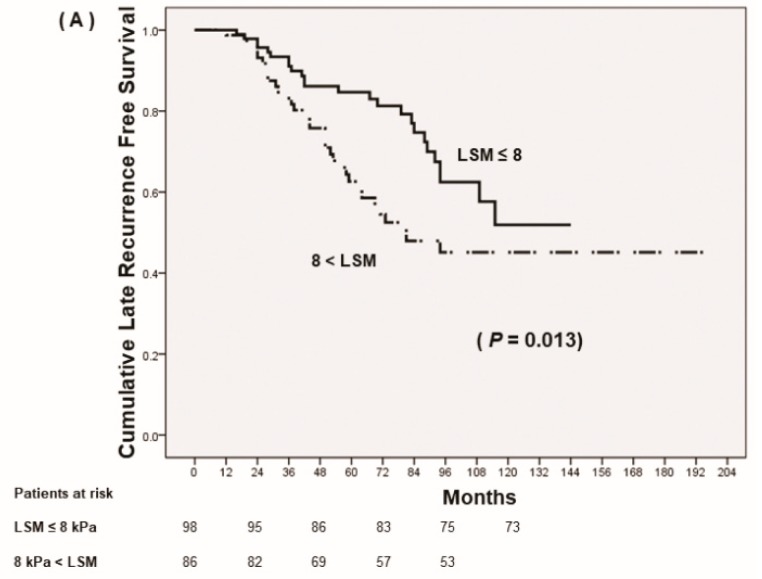

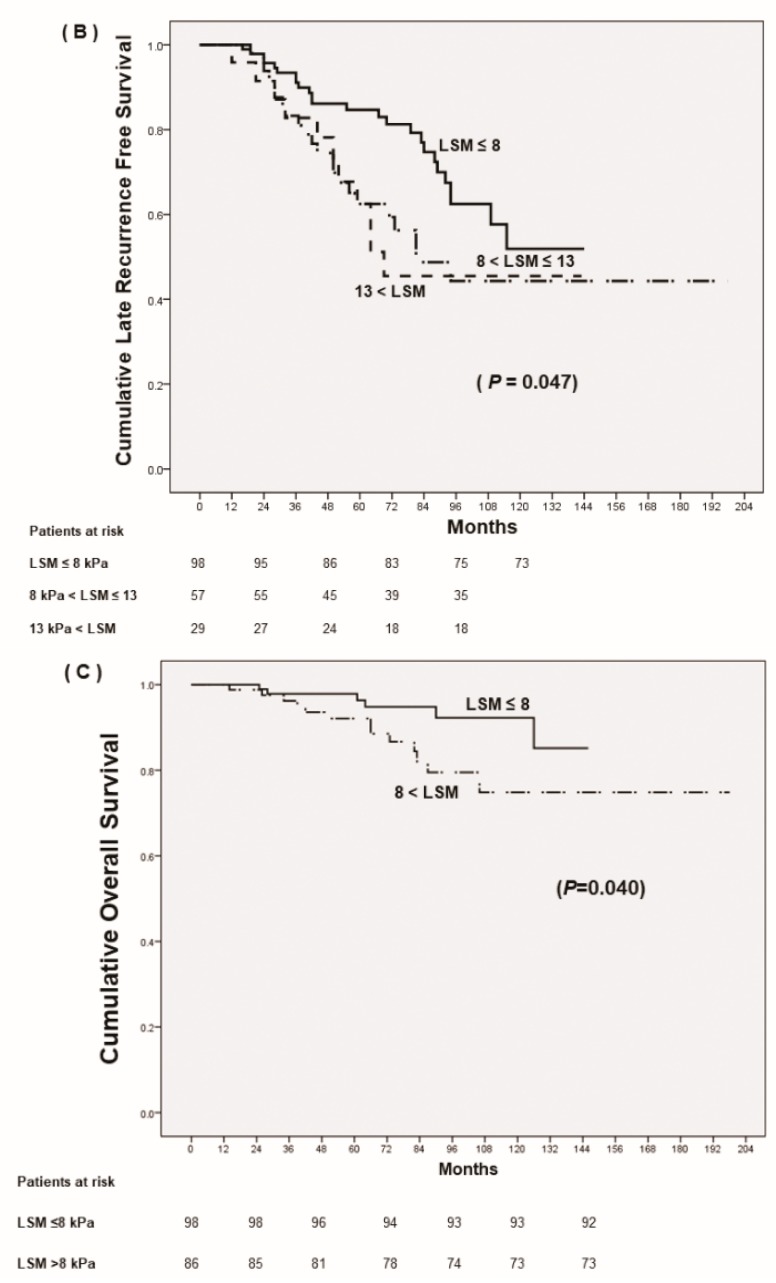
Prognosis of HCC after curative resection according to follow up-liver stiffness measurements (LSM). (**A**) Cumulative late recurrence-free survival based on stratified LSM, ≤8 kPa vs. >8 kPa. (**B**) Cumulative late recurrence-free survival based on stratified LSM, ≤8 kPa vs. 8 kPa < LSM ≤13 kPa vs. >13 kPa. (**C**) Cumulative overall survival.

Although all the patients were under antiviral therapy, achieving low fibrotic burden (LSM ≤ 8 kPa) after antiviral therapy predicted reduced late recurrence and increased recurrence-free survival rates, which was pathologically confirmed at the time of liver resection (*p* = 0.013) (Figure 2A). Patients with LSM >13 kPa and suggested to have liver cirrhosis by LS criteria demonstrated RFS rates similar to those of patients with LSM under 13 kPa but greater than 8 kPa (*p* = 0.936) (Figure 2B). However, the RFS rates of these patients were significantly lower than those of patients with LSM ≤ 8 kPa (*p* = 0.047).

Early recurrence (≤12 months) was observed in 13 patients (13/184, 7.1%). Multiple HCC nodules (HR, 6.010; 95% CI: 1.809–19.971; *p* = 0.003) and having microvascular invasion (HR, 2.779; 95% CI: 1.126–6.856; *p* = 0.027) suggested an increased risk of early recurrence, whereas preoperative low APRI score (HR, 0.169; 95% CI: 0.058–0.489; *p* = 0.001) predicted a lower risk of early recurrence (Table 4). Having low LSMs (≤8 kPa) during the follow-up period was not associated with early recurrence.

### 3.3. Follow-up LSM as a Predictor of Mortality

Among the patients with HBV-related HCC at BCLC 0–A with advanced fibrosis that underwent curative liver resection, the 1-, 2-, 3-, 5- year overall survival rates were 99.5%, 95.1%, 90.6% and 69.9%, respectively. The multivariate regression analysis for determining independent factors for mortality is shown in Table 5. Although patients had pathologically confirmed liver cirrhosis at the time of HCC occurrence, achieving low LSM (≤8 kPa) after antiviral therapy suggested decreased cumulative incidence of mortality (*p* = 0.040) (Figure 2C). In addition, not having microvascular invasion of tumor, without early tumor recurrence (≤12 months), and low APRI score at the time of tumor resection suggested decreased mortality.

## 4. Discussion

This study demonstrates that among HCC patients with pathologically diagnosed liver cirrhosis receiving under antiviral therapy after HCC resection, achieving a decreased LSM to the point where lower fibrotic burden is likely to be suggested was associated with a significantly reduced risk of late recurrence and had a beneficial effect on overall survival.

Liver cirrhosis has been recognized as the most powerful risk factor of HCC development [2,3]. Although liver cirrhosis was previously thought to be irreversible, evidence supporting the reversal of cirrhosis by eliminating the underlying cause of liver injury, such as by using antiviral therapy, has accumulated [4,5,6,7,28]. Whether the reversal of liver fibrosis by antiviral therapy would lead to a reduction in HCC occurrence, especially in those with liver cirrhosis, is still in dispute. A previous study reported that antiviral therapy failed to prevent HCC development in patients with cirrhosis [29], whereas more recent studies suggested that antiviral therapy reduced the risk of HCC occurrence even in patients with liver cirrhosis [30,31]. However, all these studied could not clearly explain whether the effect of antiviral therapy was associated with an improvement in liver fibrosis or with suppressed HBV replication. Moreover, it is not clearly delineated which patients would benefit with decreased tumor recurrence after antiviral therapy.

Sequential liver biopsy is very difficult to perform due to its invasiveness and related ethical problems. Instead, LSM by TE has been widely accepted to be well correlated with pathologic stages of liver fibrosis [32,33,34], and several studies investigated the value of LSM in predicting the risk of de novo HCC development [12,14,27,34,35]. These studies suggest that higher LSM predicted an increased risk of HCC development, and even though the cutoff values for the increased risk may still need to be settled, they were generally accepted to be >8 kPa, indicating high probability of a fibrosis stage around F3. Recently, it has been reported that LSM can be dynamically changed in both HCV and HBV infection after viral suppression or eradication, and this might result in a decreased risk of HCC development and liver-related events [18,20,21]. However, it has not been evaluated whether a reduction in LSM by antiviral therapy in cirrhotic HCC patients would result in a decreased risk of HCC recurrence, especially at a later stage after curative HCC treatment. These patients already experienced HCC development in a fibrotic liver background, and there has not been a study showing that the mere reduction in LSM by antiviral therapy would be a meaningful marker predicting changes in HCC prognosis in these patients. In our study, the results suggest that among cirrhotic HCC patients under antiviral therapy after surgical HCC treatment, achieving LSM ≤8 kPa following antiviral therapy indicated a significantly reduced risk of HCC late recurrence and might be a useful marker predicting de novo recurrence after the curative liver resection.

It has been suggested that HCC early recurrence probably represents primary metastasis from the initial tumor and is dependent on tumor factors such as tumor numbers, existence of vascular invasion, and resection margin [23,36,37]. As for late recurrence, it is likely to result from multi-centric occurrence and be associated with underlying liver disease such as high HBV DNA level, liver inflammation, and severity of liver fibrosis [38]. Although it can still be argued that reduced LSM ≤8 kPa may not necessarily indicate the pathological reversal of liver cirrhosis, low LSM can still be a reliable marker predicting favorable oncologic outcomes in cirrhotic HCC patients.

Our study has several limitations, mainly due to its retrospective nature. First, durations of antiviral therapy varied among the patients. The response to antiviral therapy in suppressing viral replication and ameliorating liver histology depends on the duration of antiviral therapy. However, all but only one patient had HBV DNA >2000 IU/mL and 156 patients (156/184, 84.8%) had undetectable HBV DNA during follow-up, suggesting that HBV replication had been effectively controlled. Whether there is a relationship between duration of undetectable HBV DNA and dynamic changes of LSM has not been adequately investigated and was beyond the scope of this study. Second, being a retrospective observational study, the point of follow-up LSM evaluation was not identical for all patients, although they were all at least 12 months after HCC resection and antiviral therapy start. Third, although all the patients had pathologically detected liver cirrhosis, a follow-up biopsy could not be performed, and decreased fibrotic burden could be estimated by achieving LSM ≤8 kPa, which is suggestive of having a METAVIR fibrosis score ≤ 2. However, it is not practical to have a liver biopsy just to evaluate liver cirrhosis regression, and achieving low LSM seems to be meaningful for predicting prognosis even in HCC patients with cirrhosis.

## 5. Conclusions

Our study suggests that low LSM (≤8 kPa) after antiviral therapy in HBV-related HCC patients with pathologically detected liver cirrhosis may be a good prognostic marker for predicting late HCC recurrence and overall survival after curative liver resection.

## Figures and Tables

**Table 1:** Demographic findings of HBV-related HCC patients with cirrhosis treated with liver resection and put under antiviral therapy.

	No Recurrence (*n* = 113)	Early Recurrence (*n* = 13)	Late Recurrence (*n* = 58)	*p-*Value
Age, mean ± SD (years)	50.4 ± 9.3	54.2 ± 7.3	55.6 ± 8.4	0.003 *
Male gender	87 (77.0%)	11 (84.6%)	49 (84.5%)	0.465
Operation type				0.329
Wedge resection	21 (18.6%)	5 (38.5%)	11 (19.0%)	
Segmentectomy	45 (39.8%)	5 (38.5%)	28 (48.3%)	
Lobectomy	47 (41.6%)	3 (23.1%)	19 (32.8%)	
Tumor size				0.622
≤3 cm	71 (62.8%)	8 (61.5%)	32 (55.2%)	
>3 cm	42 (37.2%)	5 (38.5%)	26 (44.8%)	
Tumor number				0.005 *
Single	110 (97.3%)	10 (76.9%)	51 (87.9%)	
Multiple	3 (2.7%)	3 (23.1%)	7 (12.1%)	
Microvascular invasion	42 (37.2%)	6 (46.2%)	19 (32.8%)	0.639
Edmonson grade				0.899
1	7 (6.2%)	1 (7.7%)	2 (3.4%)	
2	43 (38.1%)	6 (46.2%)	26 (44.8%)	
3	53 (46.9%)	5 (38.5%)	23 (39.7%)	
4	10 (8.8%)	1 (7.7%)	7 (12.1%)	
AFP, baseline				0.105
<200 IU/mL	81 (71.7%)	10 (76.9%)	50 (86.2%)	
≥200 IU/mL	32 (28.3%)	3 (23.1%)	8 (13.8%)	
MELD score, baseline, mean ± SD	16.2 ± 2.0	16.4 ± 1.6	16.6 ± 2.0	0.500
MELD score, follow-up, mean ± SD	16.0 ± 1.9	16.3 ± 1.8	16.1 ± 2.0	0.844
HBV DNA				0.117
<2000 IU/mL	59 (52.2%)	5 (38.5%)	21 (36.2%)	
≥2000 IU/mL	54 (47.8%)	8 (61.5%)	37 (63.8%)	
HBV DNA				0.353
<2000 IU/mL	113 (100%)	13 (100%)	57 (98.3%)	
≥2000 IU/mL	0 (0%)	0 (0%)	1 (1.7%)	
HBeAg positivity	26 (23.0%)	5 (38.5%)	15 (25.9%)	0.468
Mode of antiviral therapy				0.110
Low barrier ^†^	36 (31.9%)	5 (38.5%)	28 (48.3%)	
High barrier ^‡^	77 (68.1%)	8 (61.5%)	30 (51.7%)	
Duration of antiviral therapy, mean ± SD (months)	71.8 ± 38.2	51.3 ± 27.7	85.3 ± 31.1	0.004 *
Duration of follow up, mean ± SD (months)	74.1 ± 37.6	55.5 ± 27.2	90.2 ± 31.8	0.001 *

HBV, hepatitis B virus; HCC, hepatocellular carcinoma; AVT, antiviral therapy; AFP, alpha-fetoprotein; MELD, model for end-stage liver disease; HBeAg, hepatitis B E antigen. Continuous variables reported as median (range). Categorical variables reported as *n* (%). ^†^ Low barrier antiviral therapy included nucleos(t)ide analogues such as lamivudine, telbivudine, and adefovir. ^‡^ High barrier antiviral therapy included nucleos(t)ide analogues such as entecavir and tenofovir.; * *p* < 0.005.

**Table 2:** Changes in noninvasive parameters of fibrosis during follow-up.

Variables	Baseline	Follow Up	*p-*Value
MELD ^†^			
No recurrence	16.2 ± 2.0	16.0 ± 1.9	0.352
Early recurrence	16.4 ± 1.6	16.3 ± 1.8	0.861
Late recurrence	16.6 ± 2.0	16.1 ± 2.0	0.003 *
LSM (kPa) ^‡^			
No recurrence	12.8 ± 7.4	9.1 ± 5.6	<0.001 *
Early recurrence	12.9 ± 6.5	12.7 ± 8.0	0.866
Late recurrence	13.9 ± 6.8	11.8 ± 12.7	<0.001 *
Interquartile range (kPa)			
No recurrence	2.1 ± 2.1	1.2 ± 1.1	<0.001 *
Early recurrence	1.6 ± 1.4	1.9 ± 1.8	0.635
Late recurrence	1.9 ± 1.6	1.6 ± 1.8	0.043 *
APRI			
No recurrence	0.7 ± 0.5	0.7 ± 0.6	0.639
Early recurrence	1.2 ± 1.5	0.7 ± 0.9	0.028 *
Late recurrence	0.8 ± 0.6	0.6 ± 0.5	0.004 *
FIB-4			
No recurrence	2.3 ± 1.4	2.4 ± 1.2	0.070
Early recurrence	3.6 ± 2.8	2.5 ± 0.9	0.345
Late recurrence	2.8 ± 1.8	2.6 ± 1.5	0.760

MELD, model for end-stage liver disease; LSM, liver stiffness measurement; APRI, aspartate aminotransferase-to-platelet ratio index; FIB-4, Fibrosis-4. Variables reported as mean ± standard deviation. * *p* < 0.05; ^†^ MELD, model for end-stage liver disease calculated as follows: MELD = 10 × ((0.957 × log_e_(creatine)) + (0.378 × log_e_(bilirubin)) + (1.12 × log_e_(prothrombin time in international normalized ratio (INR))) + 6.43); ^‡^ LSM measured using transient elastography, expressed in kilopascal (kPa).

**Table 3:** Independent predictors of late recurrence after curative HCC resection in patients with pathologically confirmed liver cirrhosis under antiviral therapy.

Univariate Analysis		Multivariate Analysis	
	*p*	Hazard Ratio (95% Confidence Interval (CI))	*p*
Age at resection <45 year ≥45 year	0.003 *	3.270 (1.296–8.251)	0.012 **
Gender	0.526		
Operation type Wedge resection Segmentectomy Lobectomy	0.158		
Tumor size ≤3 cm >3 cm	0.415		
Tumor number Single Multiple	0.001 *	3.146 (1.396–7.089)	0.006 **
Microvascular invasion	0.313		
Edmonson grade ≤2 >2	0.628		
LSM, initial (kpa) ≤8 >8	0.644		
APRI, initial <1.5 ≥1.5	0.366		
FIB-4, initial <2.67 ≥2.67	0.176		
LSM, follow up (kPa) ≤8 >8	0.013 *	0.519 (0.307–0.877)	0.014 **
APRI, follow up <1.5 ≥1.5	0.378		
FIB-4, follow up <2.67 ≥2.67	0.725		
AFP, initial <200 ng/mL ≥200 ng/mL	0.182		
HBV DNA, initial <2000 IU/mL ≥2000 IU/mL	0.568		
HBeAg positivity	0.593		
Type of antiviral therapy ^†^ Low barrier High barrier	0.922		
MELD, initial	0.773		
MELD, follow up	0.425		

HCC, hepatocellular carcinoma; AVT, antiviral therapy; AFP, alpha-fetoprotein; LSM, liver stiffness measurement; APRI, aspartate aminotransferase-to-platelet ratio index; FIB-4, Fibrosis-4; MELD, model for end-stage liver disease. The initial values were assessed at the time of HCC resection and follow-up values were evaluated using the measurements obtained within 1 month before or after the last LS assessment. * *p* < 0.1, ** *p* < 0.05, ^†^ Type of antiviral therapy was categorized as low-barrier nucleos(t)ide analogues such as lamivudine, telbivudine and adefovir, and high-barrier nucleos(t)ide analogues such as entecavir and tenofovir. Please define “^a^” in the table, and cite ^†^.

**Table 4:** Independent predictions of early recurrence after curative HCC resection in patients with pathologically confirmed liver cirrhosis under antiviral therapy.

Univariate Analysis		Multivariate Analysis	
	*p*	Hazard Ratio (95% CI)	*p*
Age at resection <45 year ≥45 years	0.420		
Gender	0.176		
Operation type Wedge resection Segmentectomy Lobectomy	0.643		
Tumor size ≤3 cm >3 cm	0.318		
Tumor number Single Multiple	0.029 *	6.010 (1.809–19.971)	0.003 **
Microvascular invasion	0.099 *	2.779 (1.126–6.856)	0.027 **
Edmonson grade ≤2 >2	0.201		
LSM, initial (kPa) ≤8 >8	0.186		
APRI, initial <1.5 ≥1.5	0.005 *	0.169 (0.058–0.489)	0.001 **
FIB-4, initial <2.67 ≥2.67	0.175		
LSM, follow up (kPa) ≤8 >8	0.432		
APRI, follow-up <1.5 ≥1.5	0.942		
FIB-4, follow-up <2.67 ≥2.67	0.714		
AFP, initial <200 ng/mL ≥200 ng/mL	0.263		
HBV DNA, initial <2000 IU/mL ≥2000 IU/mL	0.163		
HBeAg positivity	0.466		
Type of antiviral therapy ^†^ Low barrier High barrier	0.181		
MELD, initial	0.712		
MELD, follow-up	0.908		

HCC, hepatocellular carcinoma; AVT, antiviral therapy; AFP, alpha-fetoprotein; LSM, liver stiffness measurement; APRI, aspartate aminotransferase-to-platelet ratio index; FIB-4, Fibrosis-4; MELD, model for end-stage liver disease. The initial values were assessed at the time of HCC resection and follow up values were evaluated using the measurements obtained within 1 month before or after the last LS assessment. * *p* < 0.1; ** *p* < 0.05; ^†^ Type of antiviral therapy was categorized as low-barrier nucleos(t)ide analogues such as lamivudine, telbivudine and adefovir, and high-barrier nucleos(t)ide analogues such as entecavir and tenofovir.

**Table 5:** Independent predictions of mortality after curative HCC resection in patients with pathologically confirmed liver cirrhosis under antiviral therapy.

Univariate Analysis		Multivariate Analysis	
	*p*	Hazard Ratio (95% CI)	*p*
Age at resection <45 year ≥45 years	0.014 *	1.76 × 10^5^ (0.00–2.235 × 10^194^)	0.957
Gender	0.039 *	0.00 (0.00–3.643 × 10^187^)	0.957
Operation type Wedge resection Segmentectomy Lobectomy	0.112		
Tumor size ≤3 cm >3 cm	0.183		
Tumor number Single Multiple	0.273		
Microvascular invasion	0.039 *	3.191 (1.188–8.568)	0.021 **
Edmonson grade ≤2 >2	0.384		
Early Recurrence (≤12 months)	0.000 *	9.416 (3.566–24.861)	0.000 **
Late Recurrence (>12 months)	0.000 *	3.366 (0.605–18.724)	0.119
LSM* initial (kPa) ≤8 >8	0.172		
LSM, follow-up (kPa) ≤8 >8	0.040 *	0.364 (0.136–0.976)	0.045 **
APRI, initial <1.5 ≥1.5	0.002 *	0.251 (0.086–0.759)	0.014 **
APRI, follow-up <1.5 ≥1.5	0.034 *	0.341 (0.097–1.203)	0.095
FIB-4, initial <2.67 ≥2.67	0.047 *	1.261 (0.345–4.613)	0.726
FIB-4, follow-up <2.67 ≥2.67	0.168		
AFP, initial <200 ng/mL ≥200 ng/mL	0.093 *	0.615 (0.071–5.350)	0.660
HBV DNA, initial <2000 IU/mL ≥2000 IU/mL	0.588		
HBeAg positivity	0.348		
Type of antiviral therapy ^†^ Low barrier High barrier	0.095 *	0.249 (0.060–1.038)	0.056
MELD, initial	0.395		
MELD, follow-up	0.932		

HCC, hepatocellular carcinoma; AVT, antiviral therapy; AFP, alpha-fetoprotein; LSM, liver stiffness measurement; APRI, aspartate aminotransferase -platelet ratio index; FIB-4, Fibrosis-4; MELD, model for end-stage liver disease. The initial values were assessed at the time of HCC resection and follow-up values were evaluated using the measurements obtained within 1 month before or after the last LS assessment. * *p* < 0.1; ** *p* < 0.005; ^†^ Type of antiviral therapy was categorized as low-barrier nucleos(t)ide analogues such as lamivudine, telbivudine and adefovir, and high-barrier nucleos(t)ide analogues such as entecavir and tenofovir.
